# Supplementary material for: Empirical methods for controlling false positives and estimating confidence in ChIP-Seq peaks
Source: BMC Bioinformatics. 2008 Dec 5;9:523. doi: 10.1186/1471-2105-9-523 (PMC2628906; doi:10.1186/1471-2105-9-523)
Supplement: Additional File 1 — A variety of html documents from the USeq web site detailing the available applications, their best usage, output file type descriptions, command line menus, etc. [file 1471-2105-9-523-S1.zip › USeqUserGuides/JavaDoc.zip!/JavaDoc/util/bio/annotation/package-use.html]

Uses of Package util.bio.annotation


|  |  |  |
| --- | --- | --- |
| **Overview** \| **Package** \| Class \| **Use** \| **Tree** \| **Deprecated** \| **Index** \| **Help** | |  |
| PREV   NEXT | **FRAMES**    **NO FRAMES**     **All Classes** |  |


---


## **Uses of Package util.bio.annotation**

| Packages that use util.bio.annotation | |
| --- | --- |
| **expr** | Classes and tools related to expression array analysis. |
| **gata.geneGlyphs** | For representing annotation, scale bars, and generic tracks. |
| **gata.main** | Main app, utility, and params for GATAPlotter. |
| **trans.anno** | For associating intervals, binding regions, and binding peaks with GFF annotation. |
| **trans.main** | Principle classes and scripts used in TiMAT2 analysis. |
| **trans.roc** | For manipulating the BAC dmel spike in data and generating ROC curves. |
| **util.bio.annotation** | For representing gene annotation. |
| **util.bio.parsers** | Parsers and extractors, fasta, quality, text, gff.... |
| **util.bio.parsers.gff** | GFF2 and GFF3 parsers. |
| **util.bio.seq** | Nucleic acid sequence manipulation. |

Classes in util.bio.annotation used by expr

****Coordinate****
  
          Base class for holding info about a genomic coordinate.

Classes in util.bio.annotation used by gata.geneGlyphs

****GeneRep****
  
          A GeneRep resentation contains a genomic sequence designating a gene broken into various segments.

****GenericFeature****
  
          Class to hold user defined gff items like CRMs, enhancers, unpublished single track notation.

****TransGroup****
  
          A TransGroup describes a product derived from a GeneGroup contains one transcript, possibly a translation, and a possibly a set of exons.

Classes in util.bio.annotation used by gata.main

****ExonIntron****
  
          ExonIntron info.

Classes in util.bio.annotation used by trans.anno

****Coordinate****
  
          Base class for holding info about a genomic coordinate.

****GeneGroup****
  
          A GeneGroup is the top organizing level of a "Gene".

Classes in util.bio.annotation used by trans.main

****ExonIntron****
  
          ExonIntron info.

Classes in util.bio.annotation used by trans.roc

****GeneGroup****
  
          A GeneGroup is the top organizing level of a "Gene".

Classes in util.bio.annotation used by util.bio.annotation

****Coordinate****
  
          Base class for holding info about a genomic coordinate.

****ExonIntron****
  
          ExonIntron info.

****GeneGroup****
  
          A GeneGroup is the top organizing level of a "Gene".

****GeneRep****
  
          A GeneRep resentation contains a genomic sequence designating a gene broken into various segments.

****ScoredCoordinate****

****StartStop****
  
          Simple start end object for sorting.

****Transcript****
  
          Transcript relevant information, mRNA.

****TransGroup****
  
          A TransGroup describes a product derived from a GeneGroup contains one transcript, possibly a translation, and a possibly a set of exons.

****Translation****
  
          Translation specific information, protein but referenced with genomic coordinates thus some
bits code for protein, some don't physically exist.

Classes in util.bio.annotation used by util.bio.parsers

****Coordinate****
  
          Base class for holding info about a genomic coordinate.

****ExonIntron****
  
          ExonIntron info.

Classes in util.bio.annotation used by util.bio.parsers.gff

****GeneGroup****
  
          A GeneGroup is the top organizing level of a "Gene".

Classes in util.bio.annotation used by util.bio.seq

****StartStop****
  
          Simple start end object for sorting.

---


|  |  |  |
| --- | --- | --- |
| **Overview** \| **Package** \| Class \| **Use** \| **Tree** \| **Deprecated** \| **Index** \| **Help** | |  |
| PREV   NEXT | **FRAMES**    **NO FRAMES**     **All Classes** |  |


---
